# NEK7 promotes gastric cancer progression as a cell proliferation regulator

**DOI:** 10.1186/s12935-021-02148-8

**Published:** 2021-08-21

**Authors:** Yi-Ke Li, Xiao-Ran Zhu, Yue Zhan, Wen-Zhen Yuan, Wei-Lin Jin

**Affiliations:** 1 Wenzhou Medical University, Wenzhou, 325035 People’s Republic of China; 2 The First School of Clinical Medicine, Lanzhou University, Lanzhou, 730000 People’s Republic of China; 3 Medical Pioneer Innovation Center, The First Hospital of Lanzhou University, Lanzhou, 730000 People’s Republic of China; 4 Department of General Surgery, The First Hospital of Lanzhou University, Lanzhou, 730000 People’s Republic of China; 5 Institute of Cancer Neuroscience, The First Hospital of Lanzhou University, Lanzhou, 730000 People’s Republic of China; 6 School of Basic Medical Science, Lanzhou University, Lanzhou, 730000 People’s Republic of China

## Abstract

**Background:**

Gastric cancer is one of the most common malignant tumors of the digestive system. However, its targeted therapy develops at a slow pace. Thus, exploring the mechanisms of the malignant behavior of gastric cancer cells is crucial to exploit its treatment. Mammalian never-in-mitosis A (NIMA)-related kinases (NEKs) are considered to play a significant role in cancer cell proliferation. However, no study has reported on NIMA family proteins in gastric cancer.

**Methods:**

Bioinformatics analysis was employed to clarify the expression patterns of NEK1–NEK11 and their effects on prognosis. The effects of NEK7 on immune infiltration and NEK7 related pathways were also analyzed. At the cell level, 5-ethynyl-2-deoxyuridine, cell cycle, and Cell Counting Kit-8 assays were utilized to clarify the effect of NEK7 on gastric cancer cell proliferation. A mouse subcutaneous model revealed the regulating effect of NEK7 on gastric cancer cell proliferation in vivo.

**Results:**

Bioinformatics analysis revealed that NEK7 is upregulated in gastric cancer and is related to poor prognosis. NEK7 is also related to T-stage, which is closely associated with cell proliferation. Further analysis showed that NEK7 was correlated with infiltration of multiple immune cells as well as gastric cancer-related pathways. Cell experiments indicated the promoting effect of NEK7 on cell proliferation, while the absence of NEK7 could lead to inhibition of gastric cancer proliferation and G1/S arrest.

**Conclusion:**

NEK7 exerts a regulatory effect on cell proliferation and is closely related to tumor immune infiltration.

**Supplementary Information:**

The online version contains supplementary material available at 10.1186/s12935-021-02148-8.

## Background

Cancer is a leading cause of death and a huge barrier to extending life expectancy worldwide. According to the statistics published on *CA* by the American Cancer Society in 2021, gastric cancer is the sixth leading cause of cancer-related deaths, and approximately one million new cases of gastric cancer were recorded, while more than 780 thousand deaths were reported in 2020 [1]. For all tumors, immortalization is the most basic characteristic, so targeting cell proliferation is an essential concept in cancer therapy for decades. However, the targeted therapy for gastric cancer develops slowly. Thus, shedding new light on the mechanism of gastric cancer cell proliferation is significant to develop new therapeutic methods and early screening.

The proliferation of eukaryotic cells, including cancer cells, relies on mitosis with a stable cell cycle. The stability of the cell cycle is maintained by many regulatory proteins, especially by kinases [2]. Abnormal expression of cell cycle-related kinases could accelerate the cell cycle and result in inappropriate proliferation. Mammalian never-in-mitosis A (NIMA)-related kinases (NEK proteins) are a group of positive regulatory proteins of the cell cycle that could regulate microtubules and promote mitosis. Expressions of NEK proteins are higher in various malignant tissues than in normal tissues. Moreover, ectopic expressions and genetic variations of NEK proteins are higher in tumor tissues. These unusual expressions of NEK proteins could lead to cell cycle dysregulation and eventually cancer [2].

In 1975, Morris investigated mitotic mutants of *Aspergillus nidulans* and found that *Aspergillus nidulans* never goes through mitosis A [3]. NIMA kinases were named after nidulans protein kinases that are encoded by the *NIMA* gene. They are a kind of serine–threonine kinases that are needed during mitosis [3, 4]. Since its discovery, 11 genetically different NIMA kinases (NEK1–NEK11) were identified in most eukaryotes, including humans. As regards the function of NEK proteins, previous studies have shown that NEK2, NEK6, NEK7, and NEK9 mainly participate in G2-M key point regulation, promote the maturity of the centrosome, and influence chromosome condensation as well as spindle formation in mitosis, while NEK1, NEK10, and NEK11 are involved in DNA damage response [5, 6].

We analyzed the expressions of NIMA kinases and their effects on prognosis synthetically and found that only NEK7 is upregulated in gastric cancer and exerted a significant effect on gastric cancer prognosis. A previous study showed that NEK7 is activated by the direct connection of NEK9 through allosteric and non-allosteric mechanisms in mitosis. Moreover, NEK7 controls phosphorylation of kinesin KIF11 and recruitment to the centrosome; as a result, the centrosome separates [7]. Thereafter, NEK7 participates in spindle assembly through phosphorylated heat shock protein NUP98 and controls cytokinesis through the regulation of motile kinesin Mklp2 as well as kinesin KIF14 [8]. Salem et al. found that lack of NEK7 could cause death in late embryonic and early postnatal periods as well as severe developmental retardation through the development and analysis of NEK7-defected mice. Meanwhile, mouse embryonic fibroblasts tended to present lagging chromosome, micronucleus formation, and cytokinesis failure [9].

Recent research presented that NEK7 was mainly connected with NLRP3 inflammasome. NEK7 could activate inflammasome NLRP3 to produce numerous polykaryocytes and apoptotic cells, which are closely related to inflammation, and then cause inflammation in the body [10, 11]. The effects of the NEK7–NLRP3 axis on diabetic retinal degeneration, systemic lupus erythematosus, and gout have already been evaluated [12–15]. Meanwhile, Eisa et al. found that the expression of NEK7 could promote cell division in cancer [16]. Zhang et al. found that NEK7 is frequently upregulated in retinoblastoma cell lines, while NEK7 knockdown by virus-mediated RNA interference could significantly inhibit cell growth as well as colony formation and arrest in the G0/G1 phase [17]. Zhou et al. also found that the expression of NEK7 is significantly higher in hepatoma cell lines than in normal liver cell lines. Furthermore, virus-mediated NEK7 silencing could inhibit the growth of hepatocellular carcinoma cell lines and tumor cells on the xenotransplantation model in immunodeficient mice [18]. Although those studies have revealed the connection between NEK7 and malignancies, NEK7 has not yet been reported in gastric cancer.

## Methods

### Cell culture

Two human gastric cancer cell lines MKN-45, MGC-803, and HEK-293 T were purchased from Beyotime Biotechnology (Shanghai, China). All cells were cultured in a medium containing 90%DMEM + 10%FBS + 1%P/S.

### Construction of NEK7 knockdown cell line

Plasmids that expressed shRNA-1 (CATTCTCGAAGAGTCATGCATAGAGATATAAAACCAGCTAA) and shRNA-2 (GAAGGCCTTACGACCGGATATGGGCTATAATACATTAGCCA) were designed. The lentiviral plasmids were constructed by the Public Protein/Plasmid Library.

After screening, shRNA-1 was used to construct stable knockdown cell lines. The lentivirus packaging kit (Gmeasy-40, Genomeditech) was then utilized.

### Protein extraction

Cells were cultured in 100-mm Petri dish until their density reached 70–90%. Radioimmunoprecipitation medium (P00103C, Beyotime Biotechnology) was used to extract total protein from cultured cells. Then, cells were boiled for 10 min after adding loading buffer (CoWin Biosciences, MA, USA).

### Reverse-transcription polymerase chain reaction (RT-PCR)

MKN-45 and MGC-803 were treated with Trizol and RNA was extracted following the manufacturer’s instructions. The RNA was dissolved in 10–100 µl of diethylpyrocarbonate-treated water, and dilution was appropriately performed for quantification. The RNA was measured by UV spectrophotometry and reverse transcribed into cDNA using a reverse transcription kit.

RNA expression was assayed by real-time PCR set to 95 ℃ for 30 s, 55 ℃ for 30 s, and 72 ℃ for 7 min and repeated for 40 circulations. Glyceraldehyde 3-phosphate dehydrogenase (GAPDH) was utilized as an endogenous control. All quantitative RT-PCR reactions were performed three times independently. The relative RNA expression levels were calculated using the 2^−△△Ct^ method.

### Western blot (WB)

Protease inhibition was used to extract total protein from cell lysis of MKN-45 and MGC-803. Bicinchoninic acid protein assay kit was used to measure protein concentration. Protein was separated by sodium dodecyl sulfate–polyacrylamide gel electrophoresis in appropriate concentration and transformed onto polyvinylidene fluoride membranes. After blocking for 1 h at 4 ℃ using tris-buffered saline with Tween^®^ 20 (TBST) brewed skim milk powder, the membrane was incubated overnight with the anti-NEK7 (ab13514, abcam, UK) antibody and anti-GAPDH (ab8245, abcam) antibody, which was diluted to an appropriate concentration. Then, after washing, the membrane was incubated with the second antibody at 4 ℃ for at least 1 h and washed by TBST three times. The anti-CDK4 (Cat No. 11026-1-AP), anti-CCND2 (Cat No. 10934-1-AP), anti-KIF3A (Cat No. 13930-1-AP), anti-AKT3 (Cat No. 21641-1-AP), and anti-PRKG1 (Cat No. 21646-1-AP) antibodies were purchased from the Proteintech Group (IL, USA). Signals were detected using a chemiluminescence system (SensiCapture imaging system, Peiqing Technology Co. LTD, China).

### 5-Ethynyl-2-deoxyuridine (EdU) to stain proliferating cells

EdU staining was utilized to analyze MKN-45 and MGC-803 cells with normal and downregulation of NEK7 expression. The EdU buffer and cell medium were mixed in a ratio of 1:1000 and added into the plate (2 ml in each well) and then incubated at 37 ℃ for 2 h. The medium was discarded, and after washing, phosphate-buffered saline (PBS) with 4% paraformaldehyde was added (2 ml each well) for cell fixation (37 ℃, 30 min). Then, the cells were permeabilized with 0.5% Triton X-100 and cultured for 10 min. The EdU staining solution was added, and the nuclei were stained with 4′,6-diamidino-2-phenylindole. The results could be visualized under a fluorescence microscope.

### Cell Counting Kit-8 (CCK-8) assay

MKN-45 and MGC-803 cells were suspended and seeded into 96-well plates. After being incubated at 37℃ for 24 h, 10 µl of CCK-8 solution (US Everbright Inc., China) was added to each well. The absorbance was measured by a microplate reader at 450 nm after being incubated for 4 h in a dark environment.

### Flow cytometry

The treated cells were collected and fixed with chilled 75% ethanol at − 20 °C overnight or longer. After ethanol was being discarded, cells were washed twice with PBS and then stained with cell cycle and apoptosis kit (C6031, UE, China) at room temperature for 30 min. Moreover, 400 μL of PBS or 1 × binding buffer was added to each tube. The selection between PBS and 1 × binding buffer was decided based on the apoptosis method and cell type. Then, cell apoptosis was analyzed immediately through flow cytometry. YF488-Annexin V was excited at 488 nm. The fluorescence emission spectrum was detected at 530 nm (fluorescein isothiocyanate channel), and the emission spectrum of the PI channel was detected at 617 nm. Cell cycle analysis was performed on the flow cytometry (FACS LSRII, BD Bioscience, China).

### Animal studies

shNC and shNEK7 cells were collected and suspended in pre-cooled PBS and subcutaneously injected into the mice (1 × 10^7^/100 µl per mouse). Twelve mice were used in total, and the negative control (n = 6) and experimental (n = 6) groups included randomly selected mice (room condition: temperature 20–26 ℃, relative humidity 40–70%, light and darkness alternate every 12 h) The mice were sacrificed at the end of the experiment (day 26). The removed tumors were used in immunohistochemistry (IHC) staining and WB. The experiments were approved by the Ethics Committee of the First Hospital of Lanzhou University (LDYYLL2021-179).

### Bioinformatics analysis and statistics

The differential gene expression in tumor and normal tissues and the correlation between protein expression and clinical prognosis of patients with gastric cancer were analyzed by GEPIA (http://gepia.cancer-pku.cn/) tool. The correlation analysis between NEK7 expression and immune infiltration level was performed using TIMER 2.0 (http://timer.cistrome.org/). GraphPad Prism 8.0 software (GraphPad Software Inc., La Jolla, CA) was also used to analyze the results. As for the quantitative PCR assay, the relative RNA expression levels were calculated using the 2^−△△Ct^ method (*p < 0.05, **p < 0.01, NS, not significant).

## Results

### NEK7 is highly expressed in gastric cancer cells and may be implicated in poor prognosis

**Fig. 1:**
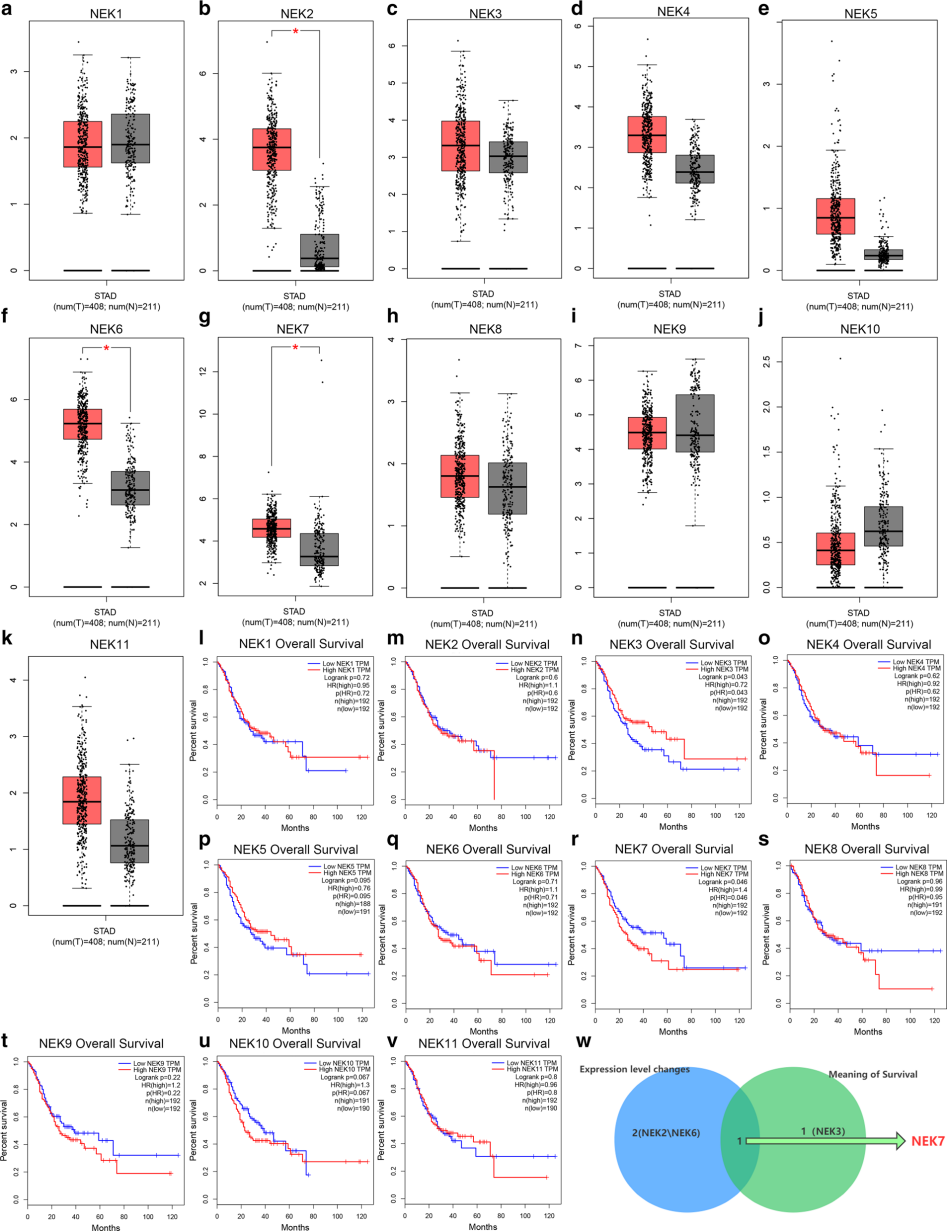
The expression levels of NEKs and their effect on prognosis. **a**–**k** Expression of NEK1–NEK11 in tumor and normal tissues (red boxes, tumor; gray boxes, normal tissue). **l**–**v**. Correlation among the expressions of NEK1–NEK11 and OS, PPS, and FP. w. The Venn diagram shows the intersection between the expression level changes and survival. *FP* first progression, *OS* overall survival, *PPS* post-progression survival

We synthetically analyzed the expression of NIMA kinases (NEK1–NEK11) (Fig. 1a–k) in gastric cancer tissues and normal tissues. The correlation between NEK protein expression and clinical prognosis of patients with gastric cancer was also analyzed (Fig. 1l–v). The results showed that only the expression level of NEK7 was upregulated in gastric cancer (|LogFC|> 1, p < 0.01 was significant) and predicted poor survival prognosis (Fig. 1w).

### NEK7 is related to gastric cancer staging

**Fig. 2:**
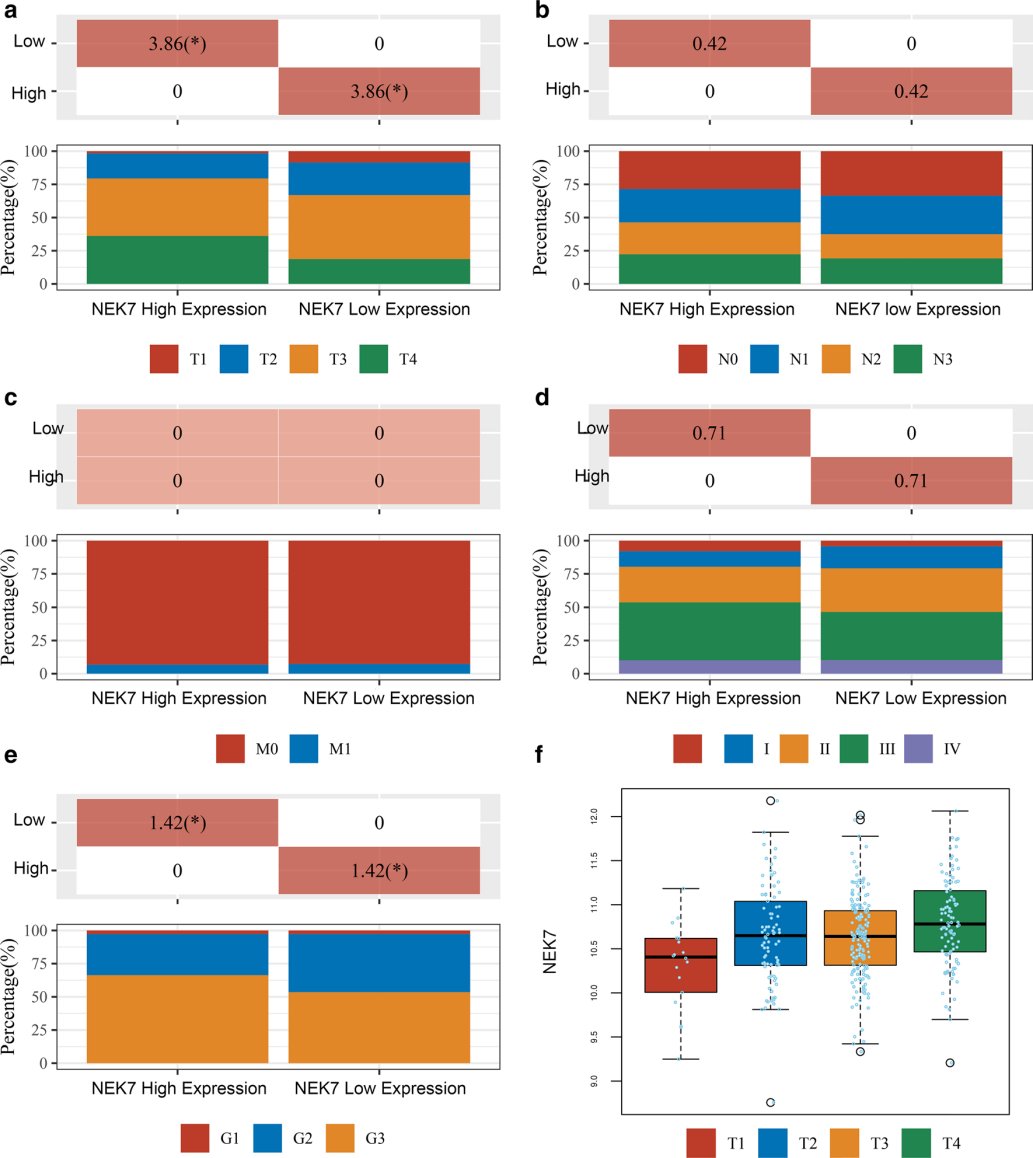
NEK7 expression effects the TNM staging and pathological grading. **a**–**d** The stacked diagram shows the percentage of each stage of the TNM staging at different NEK7 expression levels. The horizontal axis represents the samples with high or low NEK7 expressions, and the vertical axis represents clinical information contained in the corresponding grouped samples analyzed with p-value by chi-square test. For significance, the value is -log10 (p-value). *Significant difference (p < 0.05). a Percentage of pT staging. **b** Percentage of pN staging. **c** Percentage of pM staging. **d** Percentage of pTNM staging. **e** The stacked diagram shows the percentage of pathological grading at different NEK7 expression levels. **f** The box plot shows NEK7 expression levels in samples of different pT stages

We obtained RNA-seq (RNA-sequencing) data and the corresponding clinical information of 375 gastric cancer samples in The Cancer Genome Atlas Stomach Adenocarcinoma (TCGA-STAD) dataset to analyze the relationship between NEK7 expression level and clinical–pathological grading (Fig. 2e) and staging (Fig. 2a–d). The expression level of NEK7 in the late pT stage is higher than that in the early stage (Fig. 2f). The results indicated that the high expression of NEK7 may be implicated in the late T stage and high pathological grade. Complete clinicopathological information is listed in Table 1.

**Table 1:** Patients’ clinicopathological information

Characters	NEK7-high	NEK7-low	P-value
Status			
Alive	103	125	
Dead	85	62	0.022
Age			
Mean (SD)	65.7 (10.3)	65.9 (11.1)	
Median [MIN, MAX]	67 [35, 90]	67.5 [39, 90]	0.856
Gender			
Female	71	63	
Male	117	124	0.474
Race			
Asian	28	46	
Black	6	5	
White	130	108	
Islander		1	0.04
pT_stage			
T1	1	4	
T1b	2	10	
T2	22	36	
T2a	6	3	
T2b	6	7	
T3	78	90	
T4	18	12	
T4a	33	13	
T4b	14	10	
TX	8		
T1a		2	0.003
pN_stage			
N0	50	61	
N1	44	53	
N2	42	33	
N3	12	14	
N3a	26	16	
N3b	1	5	
NX	12	4	0.058
pM_stage			
M0	165	165	
M1	12	13	
MX	11	9	0.888
pTNM_stage			
IA	3	11	
IB	19	18	
II	12	15	
IIA	11	24	
IIB	27	22	
IIIA	34	26	
IIIB	24	28	
IIIC	24	11	
IV	19	19	
I		2	
III		3	0.036
Grade			
G1	5	5	
G2	56	81	
G3	120	99	
GX	7	2	0.025
New_tumor_event_type			
Metastasis	31	23	
Metastasis: recurrence	3	1	
Primary	2	1	
Recurrence	17	12	
Recurrence: primary		1	0.907
Radiation_therapy			
Non-radiation	61	84	
Radiation	25	19	0.122
History_of_neoadjuvant_treatment			
No neoadjuvant	188	187	
Therapy_type			
Ancillary: chemotherapy	19	13	
Chemotherapy	63	65	
Chemotherapy: other. Specify in notes	1		
Chemotherapy:		1	
Chemotherapy: targeted molecular therapy		1	0.406

### NEK7 is associated with immune cell infiltration

**Fig. 3:**
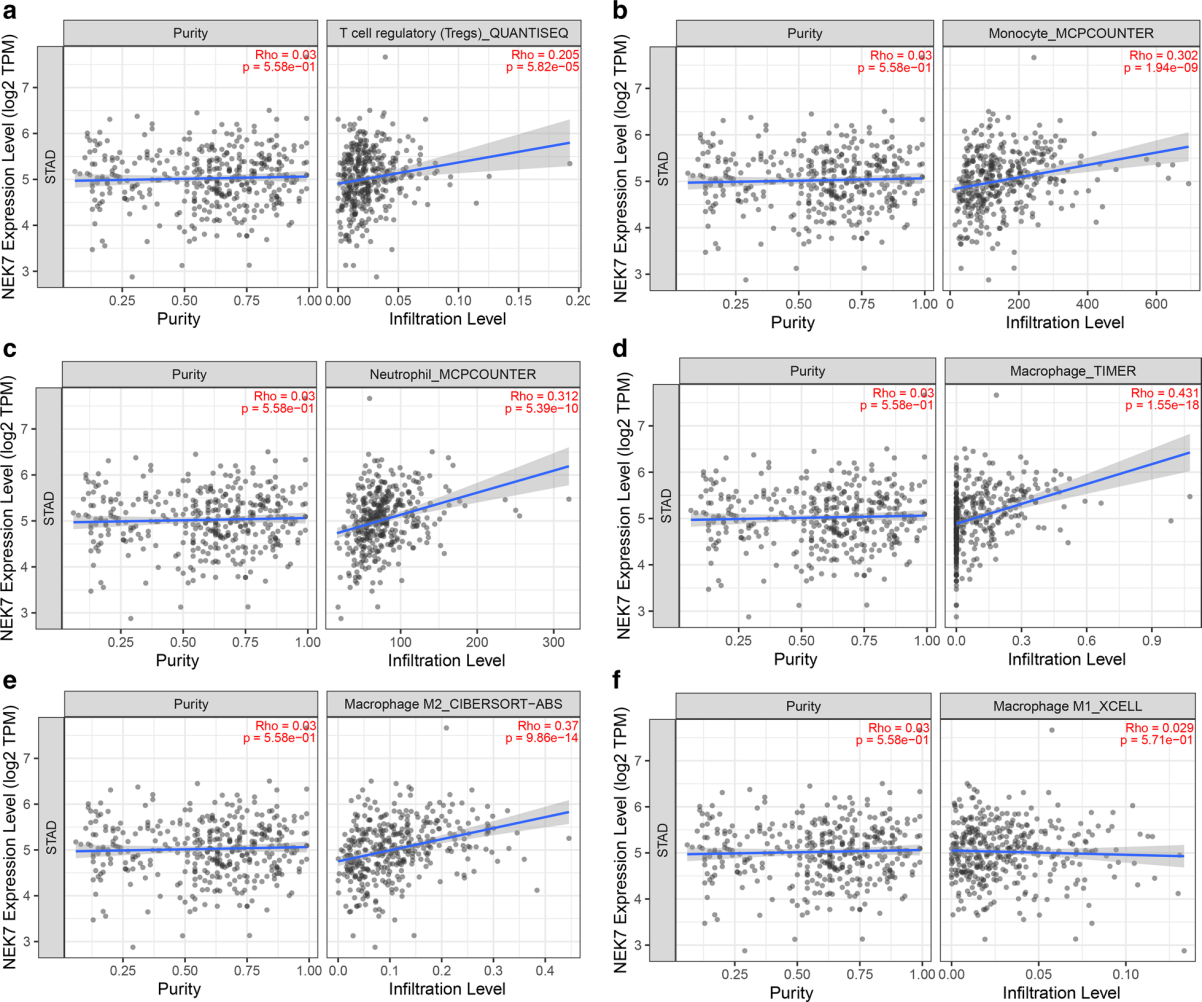
The correlation between NEK7 and immune infiltration. **a** Tregs infiltration is positively related to the NEK7 expression level. **b** Monocyte infiltration is positively related to the NEK7 expression level. **c** Neutrophil infiltration is positively related to the NEK7 expression level. **d** Macrophage infiltration is positively related to the NEK7 expression level. **e** Macrophage M2 infiltration is positively related to the NEK7 expression level. **f** Macrophage M1 infiltration is negatively related to the NEK7 expression level

TIMER 2.0 was used to analyze the effect of NEK7 on immune infiltration. The expression of NEK7 is positively related to the infiltration of Treg cells (Fig. 3a), macrophages (Fig. 3b), monocytes (Fig. 3c), neutrophils (Fig. 3d), and M2 macrophages (Fig. 3e) in gastric cancer, even if it has no distinct correlation with infiltration of M1 macrophages (Fig. 3f). Overall, the results demonstrated that immune cell infiltration could be affected by NEK7.

### NEK7 is related to an important pathway of cell proliferation

**Fig. 4:**
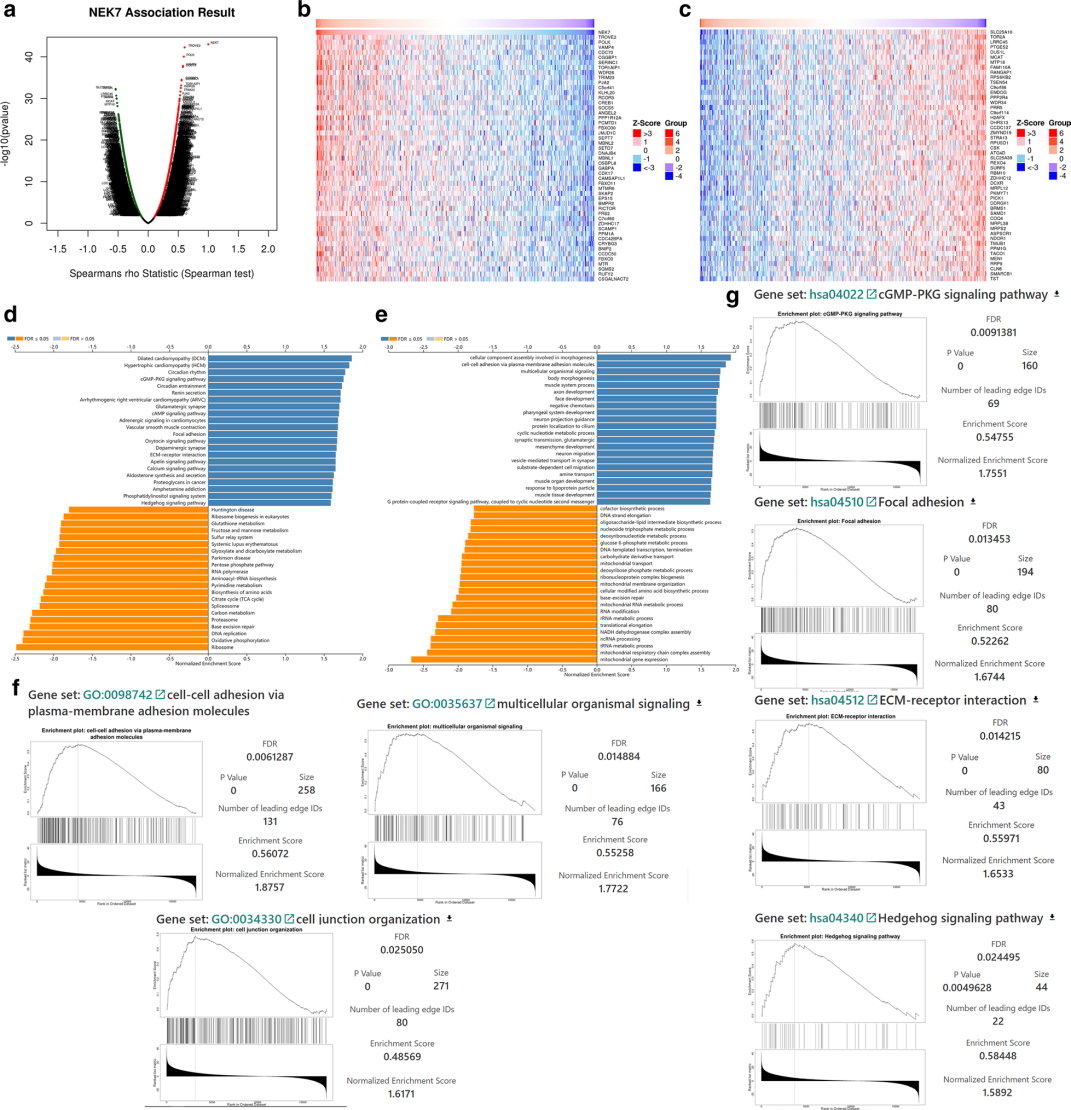
Analysis of NEK7 and relative signaling pathways. **a** The volcano plot shows the genes associated with NEK7. **b** The heat map shows the first 50 pathways positively related to NEK7. **c** The heat map shows the first 50 pathways negatively related to NEK7. **d** Kyoto Encyclopedia of Genes and Genomes (KEGG) analysis of gene pathways associated with NEK7. **e** Gene ontology analysis of gene pathways associated with NEK7. **f** Gene ontology-biological processes analysis shows pathways associated with NEK7. **g** Gene set enrichment analysis (GSEA) shows the relationship between NEK7 and cGMP-PKG signaling pathway, focal adhesion, extracellular matrix (ECM)-receptor interaction, and Hedgehog signaling pathway

By using RNA-seq data of gastric cancer from TCGA, we analyzed genetic correlation and obtained a gene map positively and negatively associated with NEK7 (Fig. 4a–c). Then, enrichment analysis on GO-BP and Kyoto Encyclopedia of Genes and Genomes (KEGG) (Fig. 4d–f) were performed. The results of the GO-BP analysis indicated that NEK7 was closely related to cell–cell adhesion via plasma membrane adhesion molecule pathway and multicellular organismal signaling pathway. Besides, NEK7 was positively related to the cell junction pathway. These results demonstrated that NEK7 was significant in regulating multicellular signaling and intracellular proliferation-related pathways. Moreover, we clarified the relationship between NEK7 and the aforementioned pathways by gene set enrichment analysis (GSEA) (Fig. 4g). Similar to the results of the KEGG analysis, GSEA showed that NEK7 was positively related to the cGMP-PKG signaling pathway, focal adhesion, extracellular matrix–receptor interaction, and Hedgehog signaling pathway. These pathways were found to be closely related to cancer progression. Moreover, using WB assay, the levels of the cGMP-PKG signaling pathway-related proteins (CCND4 and KIF3A) and Hedgehog signaling pathway-related proteins (AKT3 and PRKG1) after NEK7 administration were downregulated (Additional file 1: Fig. S1a). The results demonstrated that NEK7 downregulation could influence cGMP-PKG and Hedgehog signaling pathways through the downregulation of related proteins. The bioinformatics analysis showed the same results (Additional file 1: Fig. S1b).

### NEK7 could promote proliferation of gastric cancer cells in vitro

**Fig. 5:**
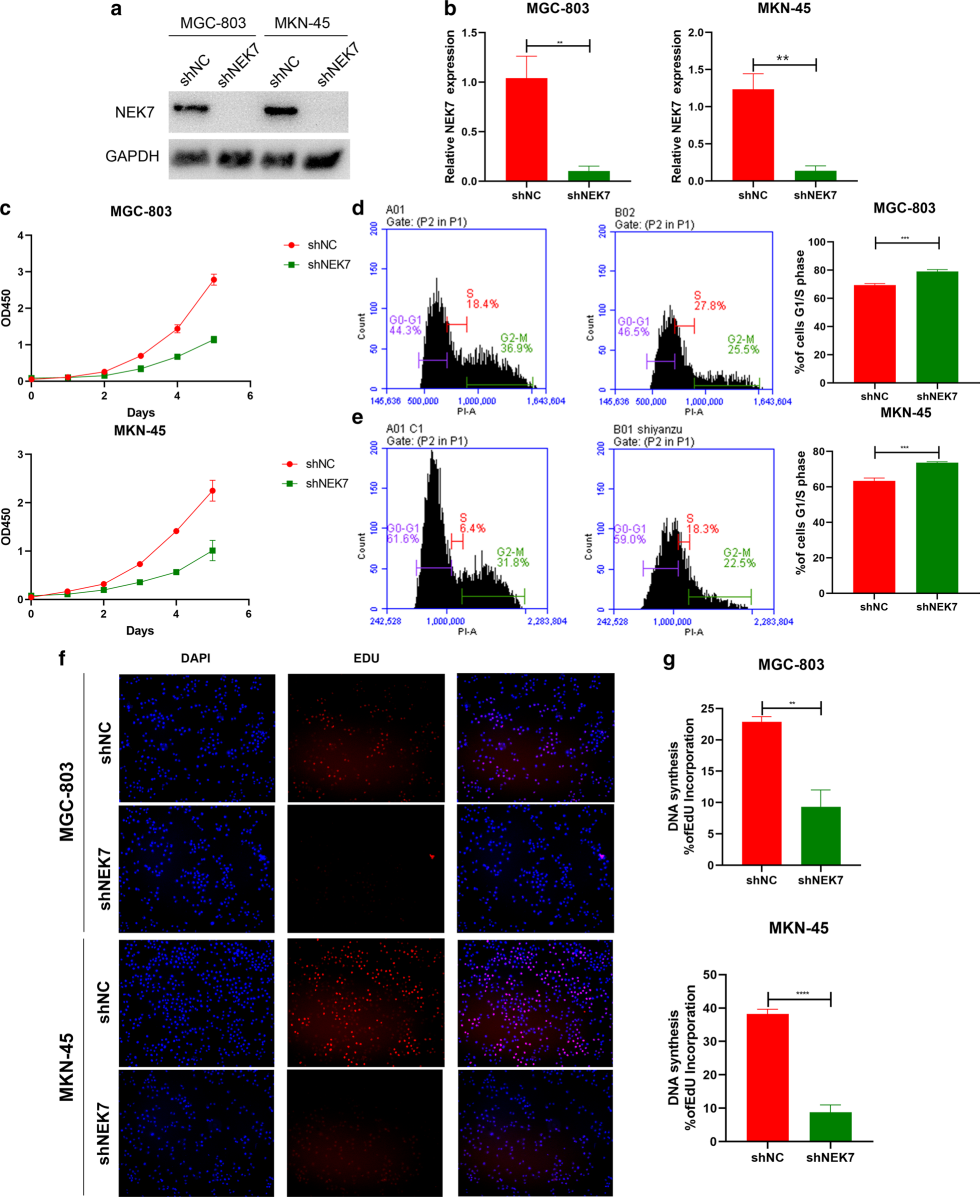
The effect of NEK7 on cell cycle. **a**, **b** The shRNA-mediated NEK7 repression and NEK7 overexpression were confirmed by western blot assay and quantitative reverse-transcription polymerase chain reaction (qRT-PCR) after lentivirus infection in the MGC-803 and MKN-45 cells. **c** Cell Counting Kit-8 (CCK-8) assay compares the OD450 values of shNEK7 cells and negative control over time. **d**, **e** The result of flow cytometry shows the effect of NEK7 on the cell cycle. **f**, **g** 5-Ethynyl-2-deoxyuridine (EdU) assay and its statistical result show the effect of NEK7 on cell proliferation

NEK7-downregulated in vitro gastric cancer models were established based on MGC-803 and MKN-45 cells. WB and quantitative RT-PCR analysis were conducted to detect the effect of NEK7 silencing (Fig. 5a, b). Then, CCK-8 assay (Fig. 5c), flow cytometry (Fig. 5d, e), and EDU assay (Fig. 5f, g) were utilized to investigate the effect of NEK7 on gastric cancer cell proliferation. The results showed that downregulation of NEK7 could inhibit proliferation of gastric cancer cells, reduce the proportion of neoplastic gastric cancer cells, and lead to cell cycle G1/S arrest. Besides, we detected the expression levels of cell cycle-related proteins such as CDK4 and CCND2 with NEK7 downregulation through western blot (WB) assay (Additional file 1: Fig. S1a). The results showed that CCND2 was downregulated, but CDK4 expression was not significantly different. The bioinformatics analysis by gene expression profiling interactive analysis (GEPIA) showed the same results (Additional file 1: Fig. S1b) The aforementioned results show that NEK7 could promote gastric cancer cell proliferation.

### NEK7 could promote gastric cancer proliferation in vivo

**Fig. 6:**
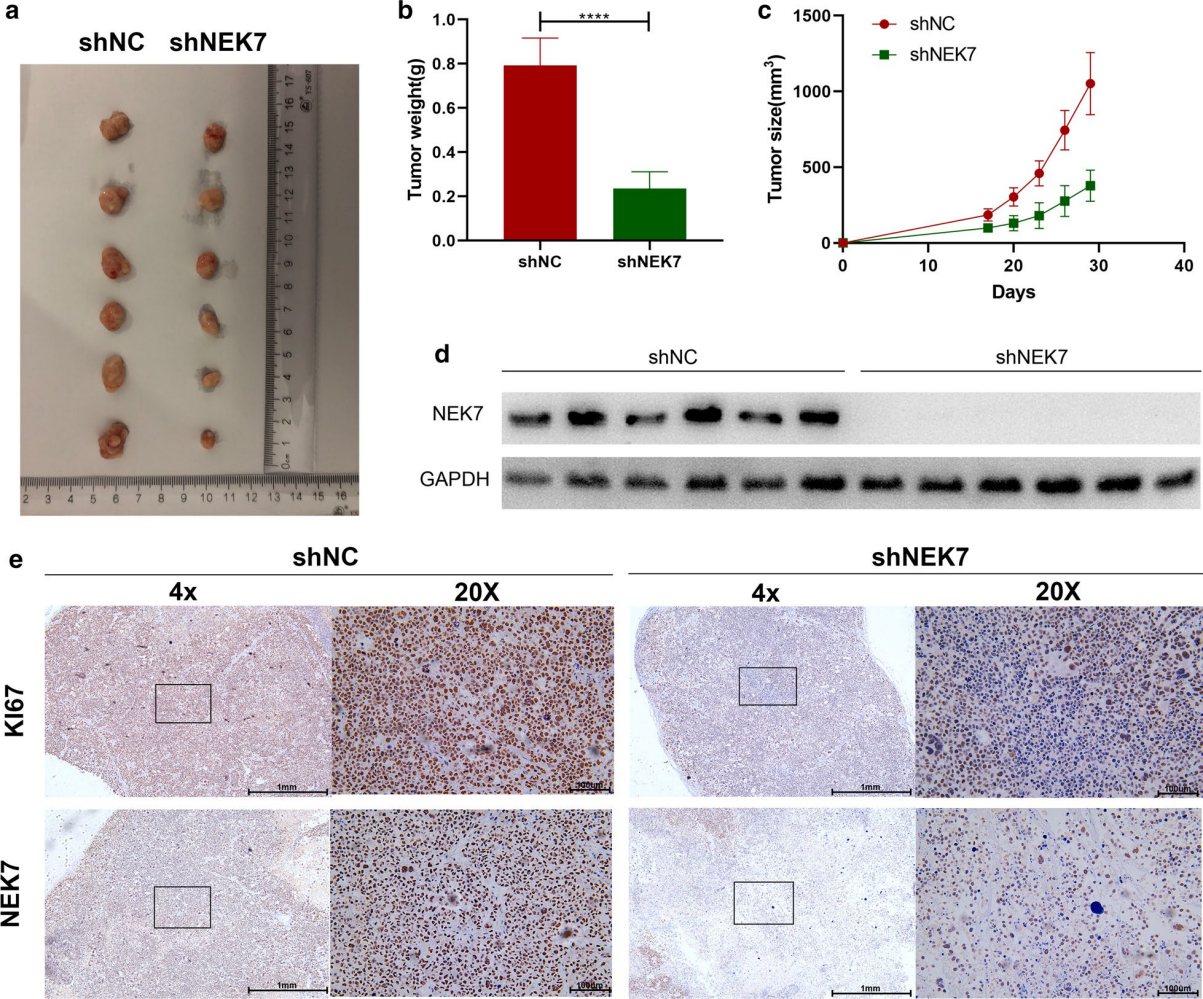
The tumor inhibition caused by absence of NEK7 in vitro. **a**–**c** NEK7 knockdown effectively suppressed subcutaneous tumor growth of gastric cancer in nude mice, and the tumor weight and size were quantitatively analyzed. **d** Western blot assay confirmed the expression of NEK7 in the subcutaneous tumor. **e** IHC (NEK7) stained and IHC (KI67) paraffin-embedded sections obtained from the MKN45-shNC and MKN45-shNEK7 subcutaneous tumors

Further, we focused on MKN45 cells and injected MKN45-shNC and MKN45-shNEK7 subcutaneously in mice (Fig. 6a). The growth of the subcutaneous tumor was monitored (Fig. 6b, c). The mice were sacrificed and dissected after 26 days. The removed subcutaneous tumors were weighed. Then, WB was performed to detect the expression level of NEK7 (Fig. 6d). Moreover, IHC was utilized to detect the expression levels of NEK7 and MKI-67 (Fig. 6e). The results indicated that the tumor volume and mass of the experimental group injected with stable MKN45-shNEK7 cells were significantly lower than those in the control group injected with MKN45-shNC cells. WB and IHC showed that the NEK7 expression levels of the experimental group were significantly lower than those of the control group. In brief, these results demonstrate that NEK7 could promote gastric cancer cell proliferation in vivo. Ultimately, NEK7 could promote gastric cancer cell proliferation both in vitro and vivo.

## Discussion

New cases of gastric cancer are increasing, and gastric cancer ranks fifth among malignancies and fourth as regards mortality worldwide. Moreover, the development of targeted therapy in gastric cancer is more limited than those in non-small cell lung cancer, chronic myelogenous leukemia, and liver cancer. Although the HER-2 targeted pathway, vascular endothelial growth factor pathway, and immune checkpoints have been widely used, the overall prognosis of patients with gastric cancer has not been revolutionized. Cell proliferation is a basic strategy of targeted therapy, so its mechanism and relationship with immune regulation are clinically worth exploring.

NEKs are a group of proteins whose domains are identical to NIMA. Some NEKs were related to tumor progression, such as breast cancer and colorectal cancer. In this study, the results of the bioinformatic analysis showed that among NEKs, the expressions of NEK2, NEK6, and NEK7 are upregulated, whereas NEK3 and NEK7 are related to the poor prognosis of gastric cancer. Thus, NEK7 was selected in this study as it shows differences in both expression levels and effects on prognosis.

The cGMP-PKG and Hedgehog signaling pathways could influence tumor cell fate determination and are closely related to the development of tumors [19–21]. According to Xiang et al., the cGMP-PKG pathway is related to gastric cancer caused by *Helicobacter pylori* [22]. Besides, Lv et al. showed that the cGMP-PKG pathway could enhance breast cancer stemness and metastasis [19]. The Hedgehog signaling pathway can promote tumor angiogenesis, metastasis, and stemness. Our data indicate that NEK7 participates in cancer cell proliferation and is related to the clinical stage as well as pathological grade. Moreover, NEK7 has a potential regulatory function in the cGMP-PKG pathway and Hedgehog signaling pathway.

Immune infiltration analysis shows that NEK7 is closely related to the infiltration of macrophages, especially M2 macrophage that could promote gastric cancer metastasis, cell proliferation, and tumor progression [23]. NEK7 could interact with NLRP3 and plays an important role in inflammatory response and determination of macrophage fate [24]. We hold the view that NEK7 could promote gastric cancer progression through not only regulation of cancer cell proliferation directly but also cell interaction, which could regulate immune infiltration and changes in immune cell subsets. In addition, GO and KEGG analyses indicate that NEK7 has a close relationship with several intercellular and matrix-related signaling pathways.

Ultimately, we revealed how NEK7 promotes gastric cancer proliferation and analyze the mechanism of promoting the progression of gastric cancer (Additional file 2).

## Supplementary Information

**Additional file 1: Fig. S1**. NEK7 effect on relative signaling pathway-related proteins. a. Expression levels of cell cycle-related proteins (CDK4 and CCND2), Hedgehog signaling pathway-related proteins (CCND2 and KIF3A), and cGMP-PKG signaling pathway-related proteins (AKT3 and PRKG1) with shNC and shNEK7 detected by western blot assay. b. Gene expression profiling interactive analysis (GEPIA) of the correlation among NEK7 and cell cycle-related proteins (CDK4 and CCND2), Hedgehog signaling pathway-related proteins (CCND2 and KIF3A), and cGMP-PKG signaling pathway-related proteins (AKT3 and PRKG1).

**Additional file 2**. Original data.

## Data Availability

The datasets used and/or analyzed during the current study can be acquired from the corresponding author upon reasonable request.

## Abbreviations

NIMA: Never-in-mitosis A
GC: Gastric cancer
EdU: 5-Ethynyl-2-deoxyuridine
CCK-8: Cell counting kit-8
STAD: Stomach adenocarcinoma
RIP: RNA immunoprecipitation
acRIP: Ac4C-RNA immunoprecipitation
TCGA: The cancer genome atlas
GEPIA: Gene expression profiling and interactive analyses
shRNA: Short hairpin RNA
qRT-PCR: Quantitative real-time PCR
WB: Western blot
IHC: Immunohistochemistry
GO-BP: Gene ontology biological process
GSEA: Gene set enrichment analysis
