# Supplementary figures and images for: NEK7 promotes gastric cancer progression as a cell proliferation regulator
Source: Cancer Cell Int. 2021 Aug 21;21:438. doi: 10.1186/s12935-021-02148-8 (PMC8379724; doi:10.1186/s12935-021-02148-8)

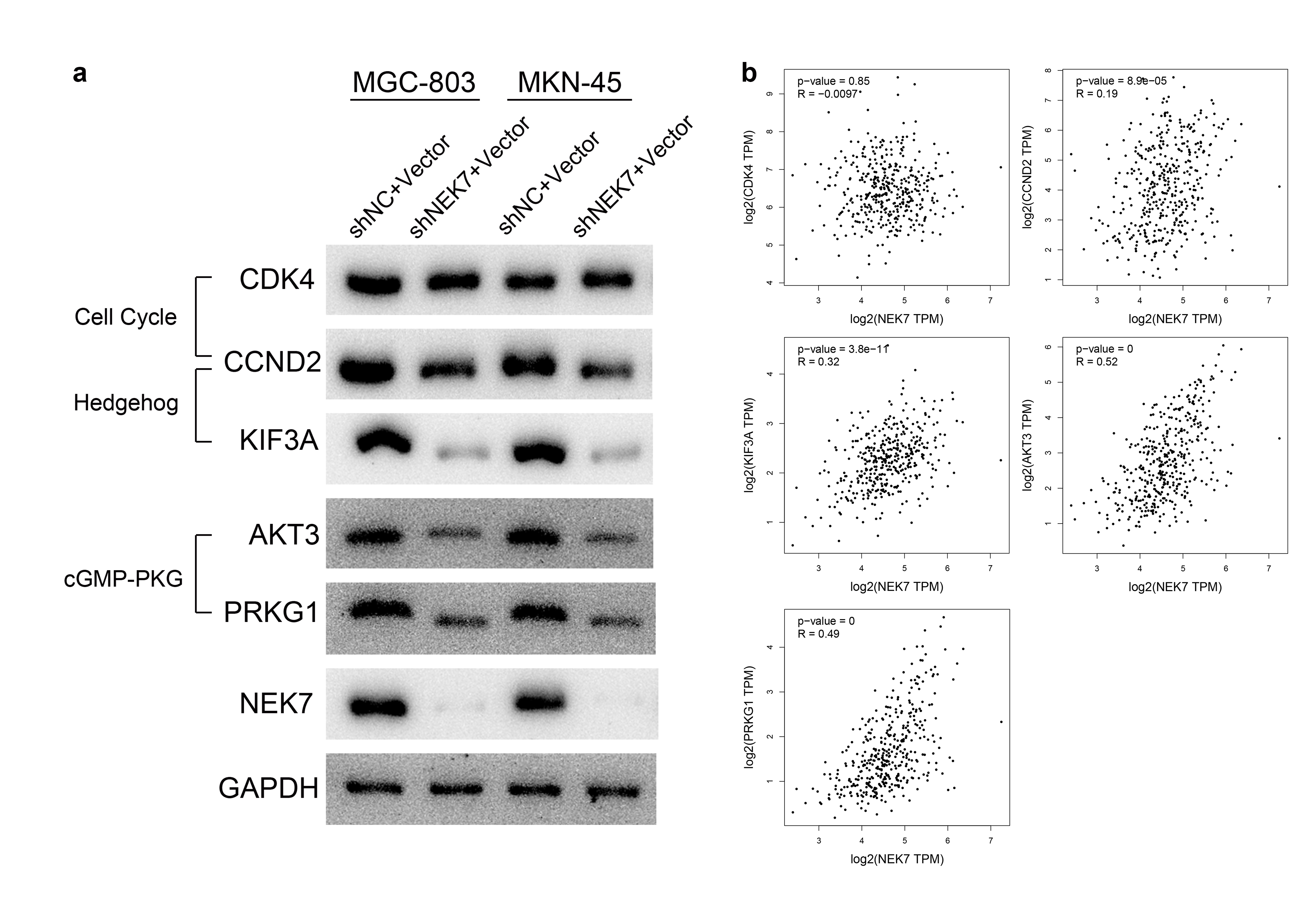

Supplement: Supplementary file 1 — Additional file 1: Fig. S1. NEK7 effect on relative signaling pathway-related proteins. a. Expression levels of cell cycle-related proteins (CDK4 and CCND2), Hedgehog signaling pathway-related proteins (CCND2 and KIF3A), and cGMP-PKG signaling pathway-related proteins (AKT3 and PRKG1) with shNC and shNEK7 detected by western blot assay. b. Gene expression profiling interactive analysis (GEPIA) of the correlation among NEK7 and cell cycle-related proteins (CDK4 and CCND2), Hedgehog signaling pathway-related proteins (CCND2 and KIF3A), and cGMP-PKG signaling pathway-related proteins (AKT3 and PRKG1). [file 12935_2021_2148_MOESM1_ESM.png]
