# Supplementary material for: NEK7 promotes gastric cancer progression as a cell proliferation regulator
Source: Cancer Cell Int. 2021 Aug 21;21:438. doi: 10.1186/s12935-021-02148-8 (PMC8379724; doi:10.1186/s12935-021-02148-8)
Supplement: Supplementary file 2 — Additional file 2. Original data. [file 12935_2021_2148_MOESM2_ESM.docx]

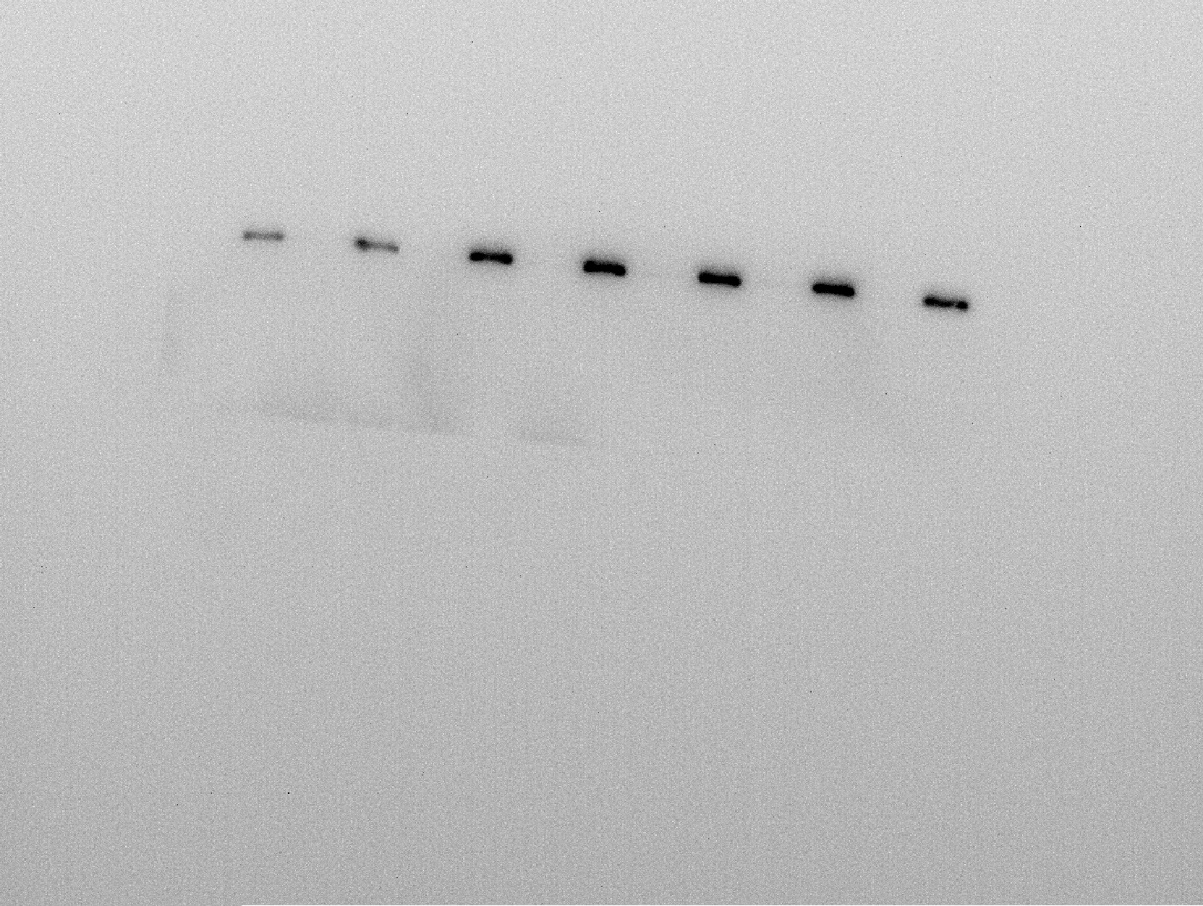


(First four) Fig.5 a, NEK7


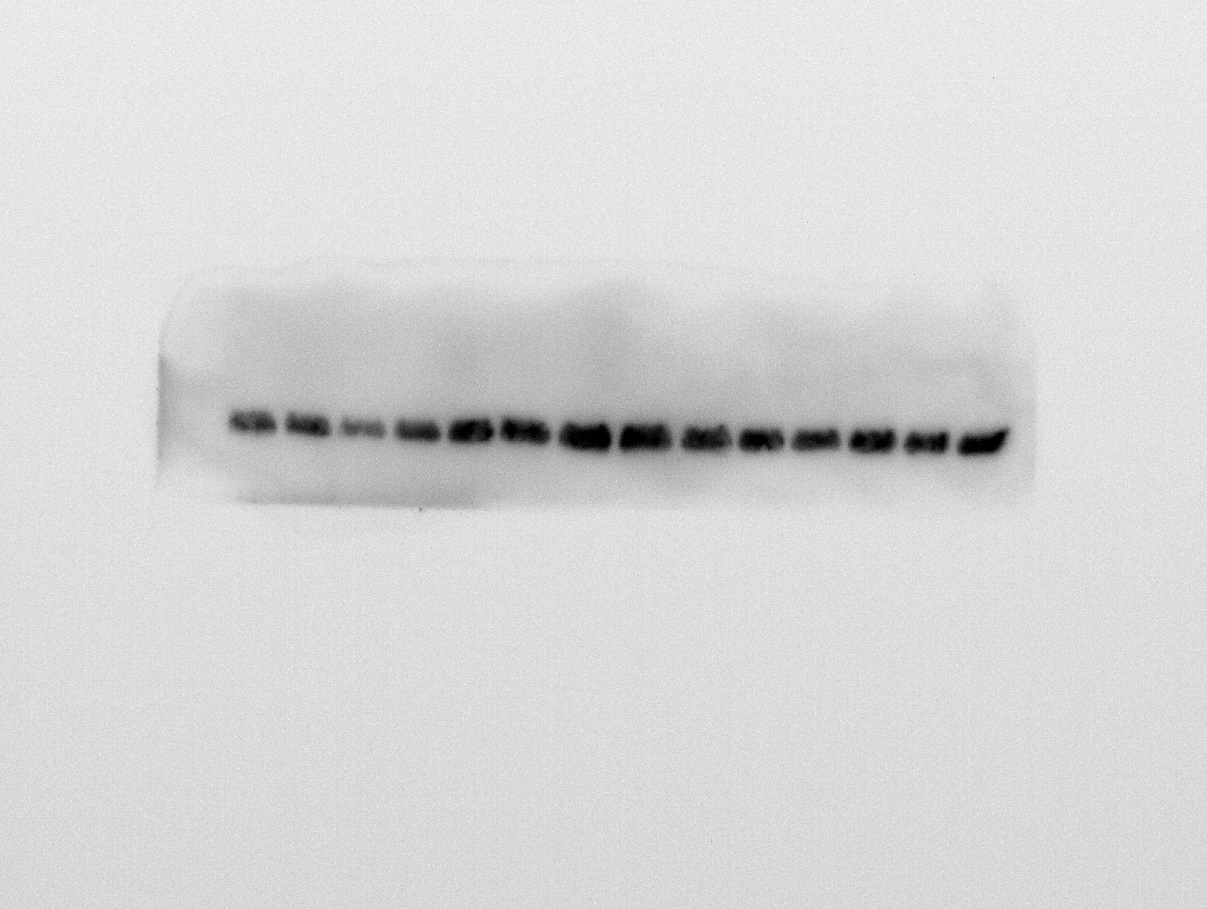


(Last four) Fig5. a, GAPDH


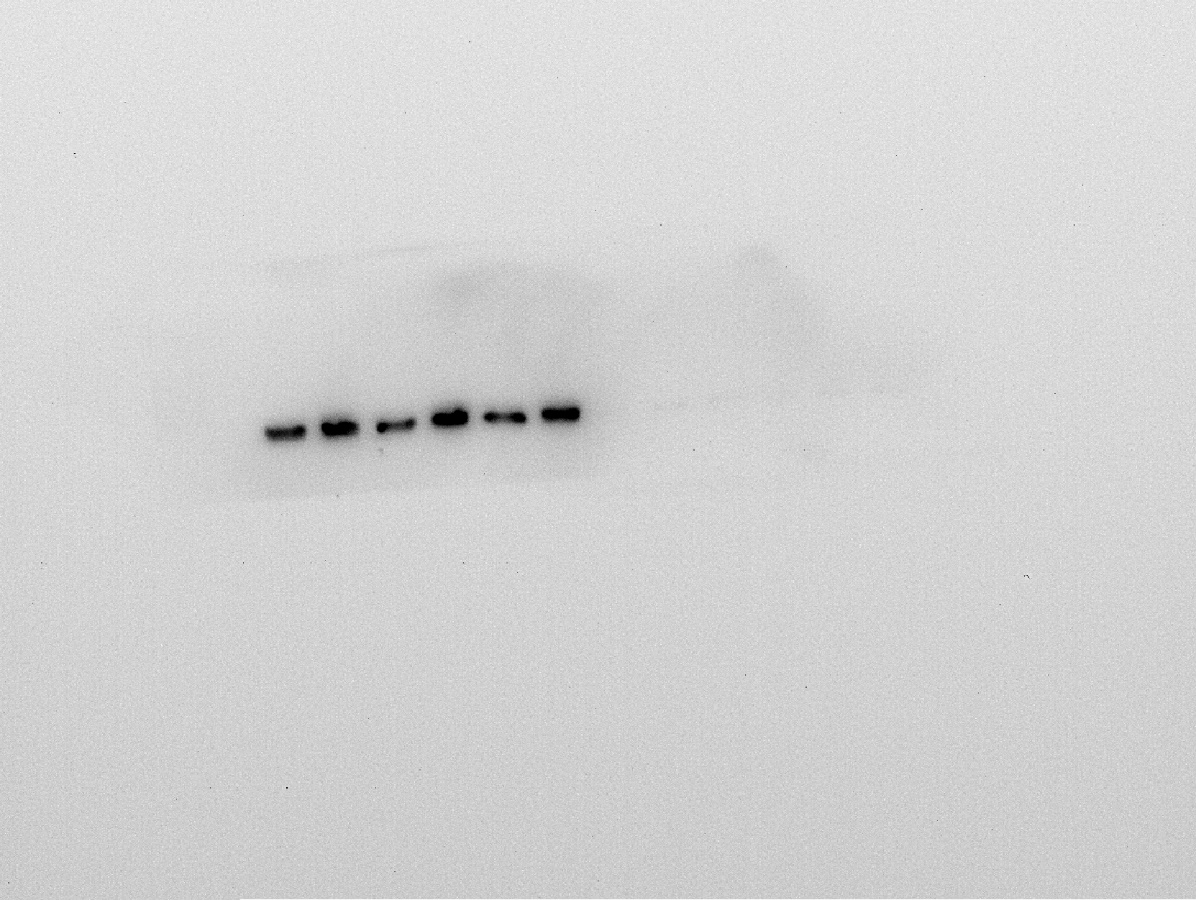


Fig 6. D, NEK7


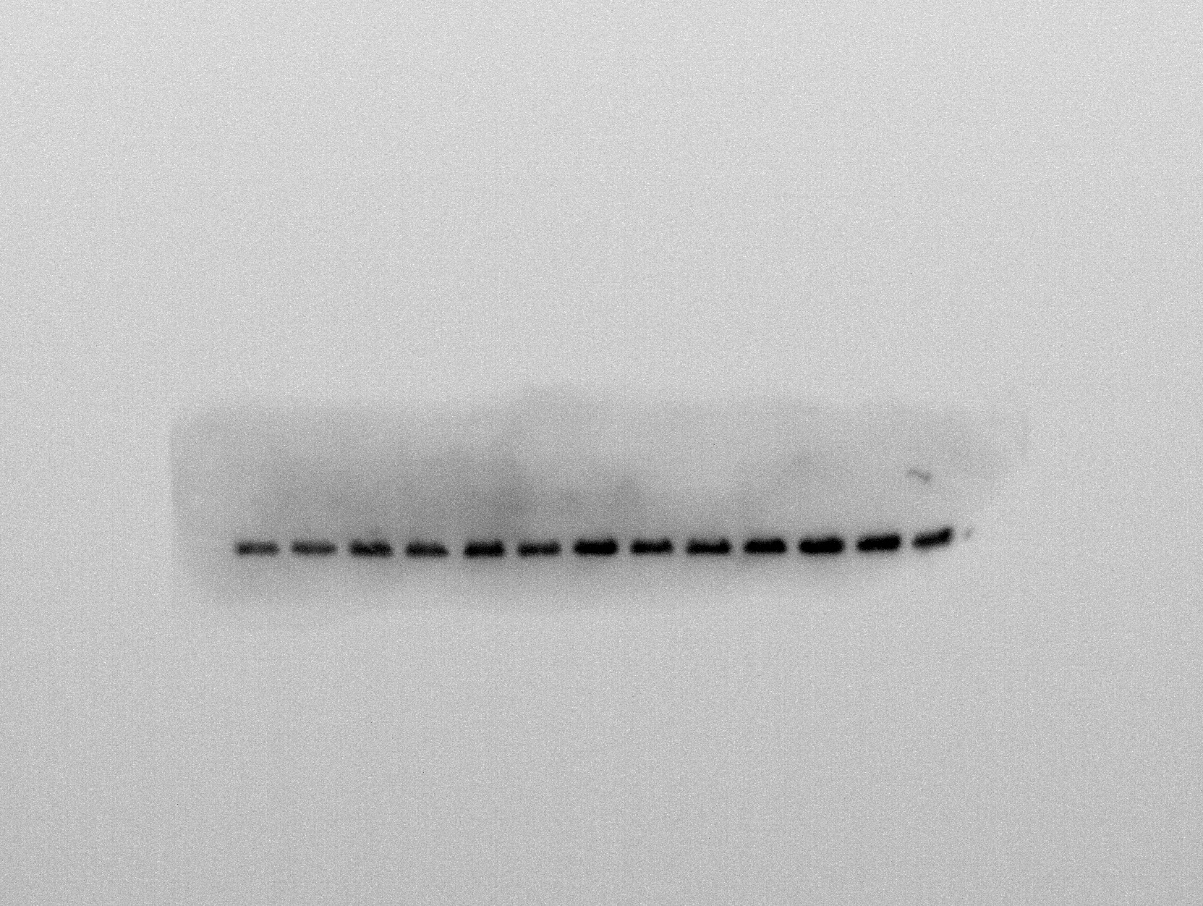


(Last 12) Fig6.d, GAPDH

The IHC photos was taken by Olympus BX51 microscope.

The Edu photos were taken by Olympus IX73 microscope.

All figures in the manuscript taken by microscope are original image.
